# Outcomes of Ventriculoperitoneal Shunt in Patients With Idiopathic Normal-Pressure Hydrocephalus 2 Years After Surgery

**DOI:** 10.3389/fsurg.2021.641561

**Published:** 2021-11-15

**Authors:** Abdul Malik Popal, Zhoule Zhu, Xinxia Guo, Zhe Zheng, Chengwei Cai, Hongjie Jiang, Jianmin Zhang, Anwen Shao, Junming Zhu

**Affiliations:** Department of Neurosurgery, School of Medicine, Second Affiliated Hospital, Zhejiang University, Hangzhou, China

**Keywords:** outcome, comorbidities, prognostic factor, ventriculoperitoneal shunt, normal-pressure hydrocephalus

## Abstract

**Objective:** To evaluate the outcomes and prognostic factors of ventriculoperitoneal shunts (VP-shunts) in patients with idiopathic normal-pressure hydrocephalus (iNPH) at 6 months and 2 years after surgery.

**Method:** We retrospectively analyzed 68 patients admitted to our institute and diagnosed with probable iNPH from January 2017 to March 2021. All patients underwent VP-shunt surgery with a programmable valve, and their outcomes were assessed via the Krauss index and modified Rankin scale (mRS) at 6 months and 2 years post-surgery. Univariate and multivariate regression analysis was performed to identify the prognostic factors.

**Results:** The mean age of the patients was 71.1 ± 8.4 (mean ± standard deviation) years. On the Krauss improvement index, 6-month follow-up results were available for 68 patients. Of these patients, 91.2% experienced attenuation of their preoperative symptoms, with a mean Krauss index of 0.58 ± 0.27, and 48 patients (70.6%) had a Krauss index ≥0.5. Two-year follow-up results were available for 33 patients; 90.9% of them had sustained improvement, with a Krauss index of 0.54 ± 0.31, and 21 patients (66.3%) had a Krauss index ≥0.5. Thirty-three patients (58%) were living independently after 2 years (mRS 0–2). The outcomes were worse for patients with multiple comorbidities. Neither an increased patient age nor a prolonged history of illness was statistically significant prognostic factors for adverse outcomes of VP-shunt surgery.

**Conclusion:** Surgical treatment was well-tolerated by patients with iNPH who received VP-shunts. Most patients experienced attenuation of their preoperative symptoms. Multiple concurrent comorbidities should be considered as adverse prognostic factors before shunt insertion in patients with iNPH.

## Introduction

Idiopathic normal-pressure hydrocephalus (iNPH) is a chronic syndrome characterized by a classic triad of gait instability, cognitive impairment, and urinary incontinence (1). Ventriculomegaly or dilated lateral ventricles (Evans index > 0.3) not explained by cerebral atrophy alone is one of the neuroradiological features in the pace of normal CSF opening pressure (2, 3). The estimated prevalence of iNPH is 1.4–2.9% among older adults (4, 5). The average age of onset is between 60 and 70 years (3, 6). The pathophysiological mechanism of iNPH remains uncertain, but with early diagnosis and treatment, the symptoms are potentially reversible via CSF shunting surgery (7). To date, the standard treatment for iNPH is to surgically divert the CSF from the ventricle to the peritoneum or other anatomical space to alleviate the clinical symptoms caused by dilated ventricles (3). Currently, the ventriculoperitoneal shunt is a commonly used shunt configuration, with a success rate of up to 80% (8–10). The use of adjustable valves enables non-invasively readjusting the pressure (3). Careful selection of patients and preoperative screening for shunt surgery are crucial for better outcomes. The degree of certainty that patients will improve after shunt surgery when their diagnoses are based solely on the clinical history and neuroimaging without supplemental testing is 50–60% (11). Applying various supplemental tests (e.g., spinal tap test, external lumbar drainage, and CSF resistance studies) offers significant advantages, and some studies reported improvement rates in up to 90% of patients (11, 12). The surgical outcome is considered being decline with time after surgery (13) despite the introduction of various prognostic factors for predicting favorable outcomes for shunt surgery (14). Nevertheless, reported data regarding clinical improvement and outcomes after shunt surgery are inconsistent owing to the application of different methods for outcome measures, differences in diagnostic approaches, varying follow-up times, substantial differences in postsurgical management, use of different shunt configurations, and the type of shunt system (5, 15–20). Several studies have shown positive outcomes after shunt surgery for patients with iNPH, with 26–90% of patients showing promising and sustained improvement after 1–10 years (16, 18, 21–24). This study was conducted to assess a single-center experience regarding the outcomes and prognostic factor associations in patients with iNPH at 6 months and 2 years after VP-shunt surgery.

## Materials and Methods

### Patients

Sixty-eight patients diagnosed with iNPH at the Second Affiliated Hospital of the Zhejiang University School of Medicine Department of Neurosurgery were included in our study. All patients were treated surgically by the same medical team. Demographic data, including patients' age, sex, initial symptoms, Evans index, symptom duration, CSF pressure on lumbar puncture, symptom severity, and initial pressure of the shunt system were recorded. The institutional ethics committee of our hospital approved this study.

### Diagnosis and Clinical Assessment

All patients were clinically examined by an interdisciplinary team (neurologist and neurosurgeon) and diagnosed via unified and standardized algorithms following the established guidelines for iNPH (25, 26). The neurologists ruled out causes of secondary ventricular enlargement and other related neurological disorders such as Parkinson's disease, Alzheimer's disease, and cerebrovascular disease. The neurosurgeon evaluated the classic triad, tap test, and neuroimaging to diagnose probable iNPH. The diagnosis was based on the mandatory presence of one aspect of the classic iNPH triad (gait disturbance, cognitive impairment, and urinary incontinence) with gradual onset in adult patients. Patients with likely iNPH symptoms underwent magnetic resonance imaging to assess ventricular dilatation. Patients with ventriculomegaly (Evans index >0.3) who displayed at least one of the three cardinal symptoms of iNPH were further assessed with a spinal tap test (spinal-TT). Before the diagnostic spinal-TT, gait was assessed with the 10-m walk test, and the following parameters were recorded: time taken in seconds to walk 10 m, step length, walking speed, and time taken to turn 180 degrees. Cognition was assessed with the mini-mental state examination (27). Urinary incontinence was evaluated based on patients' subjective feelings, from normal to severe urinary incontinence. An identical assessment was performed 24–72 h later after a spinal-TT by collecting 30–50 ml of CSF. Response to the spinal-TT was defined as the patients' gait improving by 20% in one of the measured parameters or by 10% in two parameters. A previous study showed that cognition scores improved by ≥3 points, also mentioned elsewhere (28), and patients' subjective feelings regarding urinary incontinence improved when they experienced decreased urinary urgency and frequency. Comorbidities were collected from patients' medical records The comorbidity index score (CMI) for each disease (Table 1) was calculated using a predictive tool for the shunt outcome (15).

**Table 1:** Comorbidity index introduced by Kiefer et al. (15).

**Risk factors**	**1 point**	**2 points**	**3 points**
Vascular	Hypertension Aortofemoral bypass Stent	Diabetes mellitus Peripheral vascular Vascular occlusion	
Cerebrovascular	Posterior circulation insufficiency ICA stenosis	Vascular encephalopathy TIA/PRIND	Cerebral infarct
Cardiac	Arrhythmia Valvular disease Heart failure/Stent Aortocoronary bypass Myocardial infarction		
Others		Parkinson's disease	

*TIA, transient ischemic attack; PRIND, prolong reversible ischemic neurologic deficit*.

### Shunt Surgery

Patients with a positive response to the spinal-TT underwent ventriculoperitoneal shunt surgery performed by a neurosurgeon, consistent with standardized surgical procedures (3, 29) and using an adjustable valve combined with a gravitational unit (Miethke proGAV 2.0). Postoperative neuroimaging was advised to review the appropriate catheter location and evidence of hematoma.

### Outcome Assessment

The clinical outcome at each follow-up was assessed using the Krauss index (30) and the modified Rankin scale [mRS; (31)]. For the Krauss index, post-shunt outcomes were assessed separately for each symptom: 0 = no or poor improvement, 1 = reasonable or good improvement, 2 = excellent improvement. Each patient's overall symptomatic improvement was quantified by calculating the total Krauss improvement index. To better compare the outcomes among patients, a fraction was used, with the numerator corresponding to the actual sum of improvement grades of all cardinal symptoms and the denominator corresponding to the possible maximal sum of improvement grades of the cardinal symptoms present before surgery. This method yielded a value between 0 and 1 (0: no improvement; 1: improvement of all three cardinal symptoms). For example, for patients with all three cardinal symptoms, the improvement index was calculated as follows: Krauss index = [0 – 2 (gait) + 0 – 2 (cognitive) + 0 – 2 (urinary)] improvement/6. For patients with two of the three cardinal symptoms, the improvement index was calculated as follows: Krauss index = [0 – 2 (symptom 1) + 0 – 2 (symptom 2)] improvement/4 (17, 30). Clinical follow-up was performed at the outpatient department or by calling patients or their caregiver at 6 months and 2 years post-surgery.

### Statistical Analysis

Continuous variables were analyzed using *t*-tests; categorical variables were analyzed using chi-square tests. Multivariate regression analysis was used to assess the influence of variates (age, CMI, symptom duration) on outcomes. All data analyses were performed using SPSS, IBM version 23. *P* < 0.05 was considered statistically significant.

## Results

Of the 68 patients with probable iNPH 52 were men and 16 were women, with a mean age of 71.1 ± 8.4 years. The mean symptom duration was 24.1 ± 18.4 months. The initial presenting symptom in most patients was gait disturbance. Among these patients, 51.5% exhibited all three cardinal symptoms; 92.6% presented with gait disturbance; 67.6% presented with urinary incontinence, and 60.2% presented with cognitive impairment. The mean lumbar opening pressure was 128.7 ± 36.4 mm H_2_O. At shunt insertion, the initial valve pressure was chosen based on lumbar opening pressure and the surgeon's preference. The mean initial valve pressure setting was 95.75 ± 22.63 mm H_2_O. Table 2 shows patients' baseline clinical characteristics.

**Table 2:** Baseline characteristics of 68 patients with idiopathic normal-pressure hydrocephalus.

**Characteristics**	**Value**
Total	68
**Sex**
Male	52
Female	16
Age (years)	71 ± 8.4
**Preoperative symptoms**
Gait disturbance	63
Urinary incontinence	46
Cognitive impairment	41
**Evans index**
≤ 0.4	58
>0.4	10
Symptom duration (months)	24.1 ± 18.4
**Comorbidity index**
0	12
1	18
2	11
3	9
4	11
≥5	7
**Lumbar puncture pressure (mm H**_**2**_ **O)**
<130	40
≥130	28
Preoperative mRS score	2.31 ± 1.11

*Values are presented as the mean ± standard deviation or number*.

### Postoperative Outcomes

**Figure 1:**
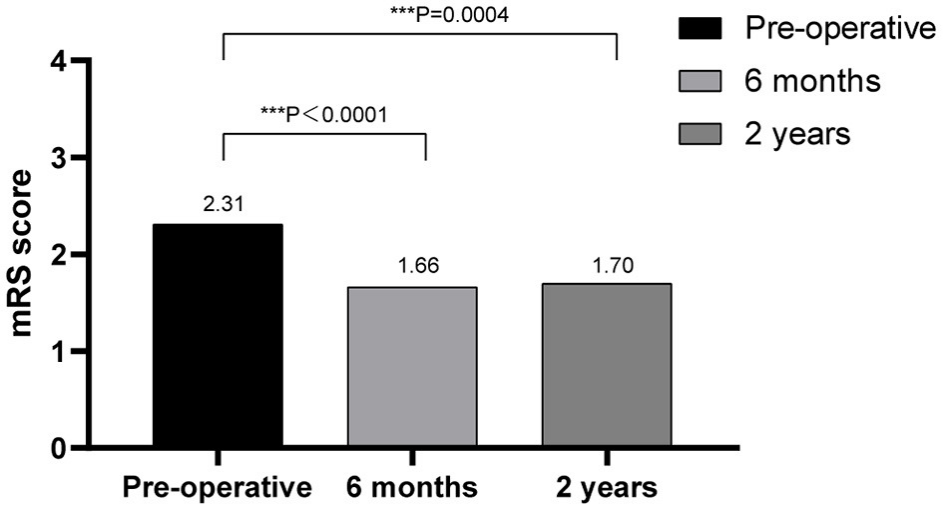
Outcomes of VP-shunt after 6 months and 2 years, comparing preoperative and postoperative mRS scores, showing significant improvement at both times. ***p < 0.001.

**Figure 2:**
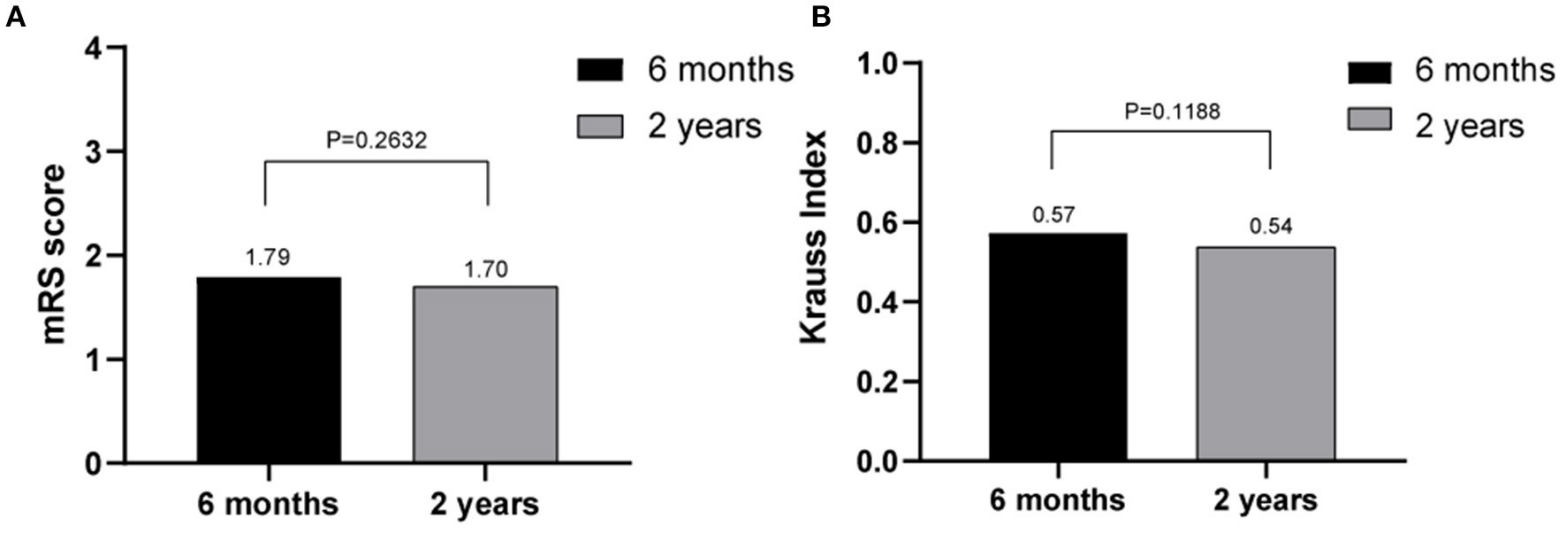
Comparison of the effects of shunt surgery at 6 months and 2 years using Krauss index scores **(A)** and modified Rankin scale scores **(B)**; no statistically significant differences were observed (*p* > 0.05).

**Figure 3:**
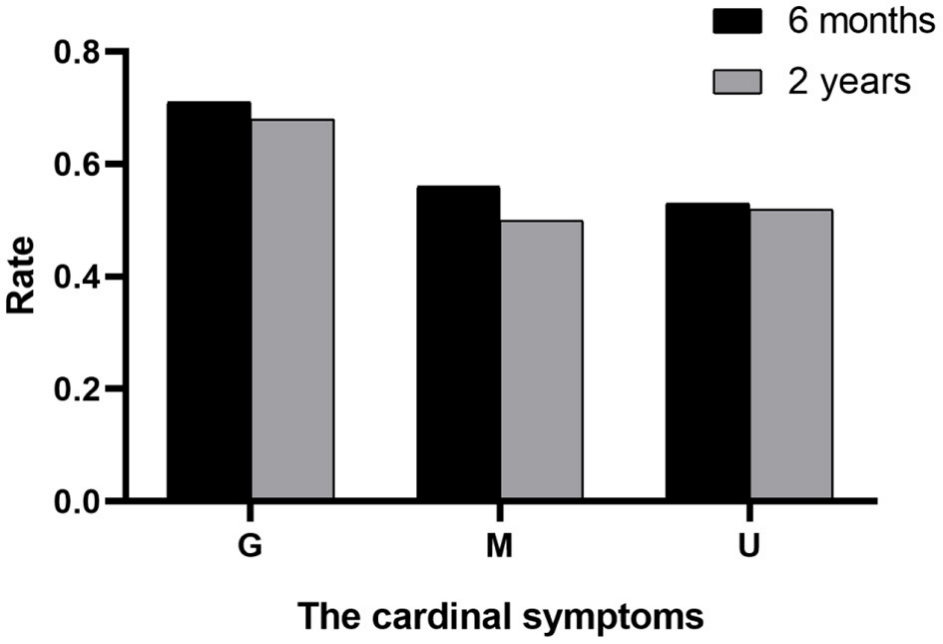
Division of the Krauss index for each improved symptom. G, gait; M, mental; U, urinary.

The outcomes of VP-shunt implantation at 6 months and 2 years post-surgery were statistically significant (*P* < 0.05; Figure 1). Six months after VP-shunt surgery (mean 6.4 ± 1.5 months), all 68 patients were available for follow-up. On the Krauss improvement index, 91.2% of patients experienced attenuation of their preoperative symptoms, with a mean Krauss index of 0.57 ± 0.27, and 48 patients (70.6%) had a Krauss index ≥0.5. The overall postoperative mRS score (mean, 1.66 ± 1.29) at 6 months significantly decreased compared with the preoperative score (mean, 2.31 ± 1.11). Of the 68 patients evaluated 6 months post-surgery, 55% showed improved mRS scores; 50% improved by one step, and 11.8% improved by two steps. The percentage of patients able to live independently (mRS score 1–2) increased from 51.5 to 73.5% at 6 months post-surgery. During the 2-year follow-up period, four patients died from causes unrelated to shunt surgery: two from pneumonia, one from a cerebral infarction, and one from an unknown cause. Thirty-three of 68 patients were available for the 2-year follow-up (mean 24.5 ± 4.7 months); 90.9% of these 33 patients had sustained improvement. The mean Krauss index was 0.54 ± 0.31; 21 patients (63.3%) had a Krauss index of ≥0.5, and 66.7% of the 33 patients were living independently after 2 years (mRS 0–2). Notably, the 6-month and 2-year outcomes after VP-shunt surgery did not differ significantly (*p* > 0.05); thus, the 6-month outcome changed minimally over the subsequent 24 months (Figure 2). Segmentation of the Krauss index for each of the three cardinal symptoms showed the most improvement in gait disturbance at both 6 months and 2 years (Figure 3). Our results showed that comorbidity had a more significant adverse effect on patients' outcomes than did patients' age and history of illness (*p* < 0.05; Tables 3, 4).

**Table 3:** Patient's characteristics and predictors of VP-shunt outcome.

**Variables**	**Krauss ≥0.5**	**Krauss <0.5**	***P*-value**
**Sex**
Male	36	16	0.526
Female	13	3	
Age	69.31 ± 9.96	73.79 ± 7.74	0.180
**Initial symptom**			0.948
Gait disturbance	35	13	
Cognitive impairment	10	4	
Incontinence	4	2	
**Evans index**			0.486
0.4	42	15	
≥0.4	7	4	
Duration of symptom (months)	25.61 ± 20.55	21.05 ± 11.19	0.805
**Comorbidity index**			0.017^*^
0	11	1	
1	14	4	
2	10	1	
3	8	1	
4	4	7	
≥5	2	5	
**Lumbar puncture pressure**			0.786
<130 mm H_2_O	28	12	
≥130 mm H_2_O	21	7	

* *Statistical significance; Cl, confidence interval 95%*.

**Table 4:** In the multivariable analysis, comorbidity was the only prognostic factor negatively influencing VP-shunt outcome.

**Variables**	***P*-value**	**HR (95% CI)**
Age	0.170	
Symptom duration of (months)	0.121	
**Comorbidity index**
≤ 3		1
>3	0.001^*^	8.786 (2.630–29.203)

*CI, confidence interval 95%*.

* *p <0.05*.

## Discussion

The efficacy of shunt surgery remains controversial, and a common perception is that the beneficial effects of shunts are short-lived (21). Junkkari et al. (32) concluded that only 43% of patients experienced a significantly improved quality of life 1 year after VP-shunt surgery. Another study reported a significant decline in improvement from 75 to 50% at 1-year post-surgery (33), and after 3 years, only one of three patients had sustained improvement in gait, one of eight had sustained cognitive improvement, and one of six had sustained improvement in urinary incontinence. A systematic review found that patients with iNPH had improvement rates as low as 29% at 3 years post-surgery (34). The heterogeneity in these shunt outcomes may be because of differences in diagnostic protocols, selection criteria, and use of various outcome measures. Standardized outcome measures for assessing iNPH are needed to reduce the inconsistency in these outcomes, and a standardized diagnostic protocol is needed to increase accuracy.

Shunt surgery in our study population generally yielded favorable results. The higher improvement rate may have been due to appropriate diagnostic protocols in line with iNPH guidelines (2, 3), careful criteria for selecting suitable candidates for shunt surgery (3), implications of supplemental predictive prognostic tests (11), and regular follow-ups, the last of which enabled identifying and fixing shunt malfunctions, which, if not diagnosed in time, would have shortened the effective life of the shunt (23). Recent studies with 1–3-year follow-ups for shunted iNPH patients have shown promising outcomes. Pujari et al. (35) found sustained improvement in 55 patients with iNPH at 3 years post-surgery for all symptoms: gait, 83%; cognition, 84%; and urinary incontinence, 84%. A European multicenter study (18) described the 1-year outcomes of VP-shunt surgery for 115 patients with iNPH: the gait improved in 77% of patients, cognition in 63%, and urinary incontinence in 66%. A recent meta-analysis (36) reported an overall improvement rate of 75% in shunted patients with iNPH. Therefore, the duration of the therapeutic effect should not be the main factor in determining whether iNPH patients are treated with shunt surgery (23). The survival rates of patients with iNPH who have shown improved gait and functional independence for daily living are similar to those of the general population, indicating that shunt surgery for iNPH both improves symptoms and normalizes survival (37). Importantly, patients with iNPH are primarily older adults, and a slight improvement in gait can improve their quality of life and reduce functional restrictions and social sequelae (38).

### Outcome Predictors

Symptoms lasting longer than 1 year, older patients, and late onset of gait disturbance or absence of gait disorder have been reported as poor predictors of shunt surgery outcomes in patients with iNPH (14, 15, 24, 39). Conversely, younger age, less severe dementia, gait as an initial complaint, and ailments with a history of <1 year are regarded as favorable outcome predictors (40, 41). Thus, an important secondary aim of this study was to assess the baseline characteristics of the study population. Inconsistent with previous studies, potential prognostic factors of outcomes, such as older age, prolonged symptom duration, initial symptoms at hospitalization, sex, and Evan's index, were not statistically significant negative predictors of VP-shunt outcomes in our series (2, 8, 19, 21, 41). Here, comorbidity alone significantly adversely affected the outcomes. We speculate that the influence of older age and a prolonged symptom history on patients' outcomes may be a pseudo-correlation mediated by comorbidity as iNPH is an adult-onset disorder and frequently associated with one or multiple comorbidities. Among our patients, 82.4% presented one or several concomitant comorbidities, which is higher than the reported mean of 44% in patients with iNPH (42). Other authors have also shown high incidences of comorbidities, and the frequencies of hypertension, diabetes mellitus, and cerebrovascular disease were higher in patients with iNPH than in control groups (43, 44). An epidemiological study found a higher frequency of hypertension in iNPH patients than in controls (52 vs. 32%) as well as an overrepresentation of type 2 diabetes mellitus of 22 vs. 12% (45). In our study, we could not show the impact of each disease alone on the outcome that would influence shunt efficacy. Consistent with other studies (15, 21, 46), we found that comorbidity substantially influenced the efficacy of shunt surgery in patients with iNPH. Hence, clinicians should consider comorbidities when counseling patients or their caregivers before shunt implantation. Patients with worse preoperative conditions and a high comorbidity burden will have higher CMI scores (>3) and less favorable outcomes from shunt surgery compared with those with fewer comorbidities. Previous studies found that patients with iNPH with comorbidities such as stroke, cerebral infarction, hypodense white matter lesions, transient ischemic attacks, Parkinson's disease, hypertension, and diabetes mellitus had less favorable responses to shunt surgery (14, 15, 47, 48). A prospective study found that 79% of iNPH patients without cerebrovascular disease (CVD) and 51% of patients with CVD had sustained improvement 4 years after shunt surgery (48). Similarly, Boon et al. (47) found a 52% improvement rate in shunted iNPH patients with CVD in contrast to 75% of patients without CVD. Kazui et al. (49) observed favorable cognitive improvements after shunt implantation in iNPH patients without hypertension compared with those with hypertension. Bech-Azeddine et al. (50) evaluated the VP-shunt outcomes in 27 patients with iNPH. After surgery, nine patients improved, eight clinically worsened, and 10 remained unchanged. All eight patients whose conditions deteriorated had Alzheimer's disease, indicating that neurodegenerative disease in patients with iNPH may affect the long-term outcomes of shunt surgery.

### Complications

A prior study reported an overall mean complication rate of 38% after shunt surgery and a shunt revision rate of 22% (34). However, a more recent literature review reported a lower pooled complication rate of 10% and a shunt revision rate of 16% (8, 36). Another study revealed that more than half of shunt revisions result from shunt malfunction, and ~50% occur in the 1st year after initial shunt surgery (35). In our series, no patients underwent shunt revision surgery within the first 2 years after the initial shunt surgery, possibly owing to our use of precise surgical techniques at the time of shunt insertion. Therefore, we believe that meticulous techniques during shunt surgery can help prevent catheter displacement, as previously indicated by Mirzayan et al. (17), as would the use of adjustable valves with the gravitational unit. Consistently, a meta-analysis found that using a programmable valve yielded a lower shunt revision rate (12%) than did use of the fixed valve (32%), and the revision rate further decreased when an antigravity component was added (36). In our patients, the neurosurgeon chose the initial pressure of the shunt (usually 20–30 mm H_2_O minus the lumbar opening pressure). However, Scholz et al. (51) recommended an initial valve pressure of 70 mm H_2_O and a gravitational unit setting such as a proSA of 200 mm H_2_O for patients shorter than 1.6 m, 250 mm H_2_O for patients with a height of 1.6–1.8 m, and 300 mm H_2_O for patients taller than 1.8 meters. The VP-shunt complication rate in this series was 11.8% (8 patients). One patient developed epilepsy, two had pain from the abdominal incision and were successfully treated conservatively, two developed an infection, and three developed subdural hematoma, which was resolved by increasing the shunt system's opening pressure. Thirty patients (44.1%) required initial shunt opening pressure readjustment to improve function.

The limitations of this study were the inability to collect missing data for some of our patients and the retrospective approach, which may be subject to selection bias. The strengths of this study were the standardized diagnostic protocol adherent to iNPH guidelines, the use of objective and subjective assessment tools, and the application of supplemental tests to identify patients most likely to respond to shunt surgery.

## Conclusion

Surgical treatment with a VP-shunt is well-tolerated by cautiously selected patients with iNPH, and most of these patients experience attenuation of their preoperative symptoms, even in the long term. In patients with iNPH, the outcomes of VP-shunt surgery did not significantly differ between 6 months and 2 years post-surgery, indicating that the outcome at 6 months remained stable for up to 24 months. Longer symptom durations and older age should not deter patients with iNPH from undergoing shunt implantation. The high burden of associated comorbidities in these patients should be considered regarding the risk-to-benefit ratio of surgical intervention. The complication rate for VP-shunt surgery is relatively low, and the success rate in these patients is high. Regular follow-up and optimization of the shunt system's function may contribute to the long-term success of shunt treatment.

## Data Availability Statement

The original contributions presented in the study are included in the article/supplementary material, further inquiries can be directed to the corresponding author/s.

## Ethics Statement

This study was reviewed and approved by the Zhejiang University School of Medicine Second Affiliated Hospital ethics board. The patients/participants provided their written informed consent to participate in this study.

## Author Contributions

AP designed the study and wrote the first draft of the manuscript. JZhu and AS contributed to the conception and design of the study. ZZhu, CC, XG, and HJ collected and analyzed the data. JZha and ZZhe contributed to substantive revision of the work. All authors strictly revised the manuscript and decided on the content of the final article.

## Conflict of Interest

The authors declare that the research was conducted in the absence of any commercial or financial relationships that could be construed as a potential conflict of interest.

## Publisher's Note

All claims expressed in this article are solely those of the authors and do not necessarily represent those of their affiliated organizations, or those of the publisher, the editors and the reviewers. Any product that may be evaluated in this article, or claim that may be made by its manufacturer, is not guaranteed or endorsed by the publisher.
